# Network controllability analysis reveals the antiviral potential of Etravirine against hepatitis E virus infection

**DOI:** 10.1128/msystems.00438-25

**Published:** 2025-08-15

**Authors:** Shabnam Ansari, Dipanka Tanu Sarmah, Rohit Verma, Kannan Chandrasekar, Shalimar, Baibaswata Nayak, Samrat Chatterjee, Milan Surjit

**Affiliations:** 1 Virology Laboratory, Center for Virus research, therapeutics and vaccines, Translational Health Science and Technology Institute, NCR Biotech Science Cluster 682813 , Faridabad, Haryana, India; 2 Complex analysis group, Computational and Mathematical Biology Center, Translational Health Science and Technology Institute, NCR Biotech Science Cluster 682813 , Faridabad, Haryana, India; 3 Department of Gastroenterology, All India Institute of Medical Sciences 28730, New Delhi, India; Boston College, Chestnut Hill, Massachusetts, USA

**Keywords:** Hepatitis E virus, connectivity map, network controllability, casein kinase 1ε, protein kinase B, Etravirine

## Abstract

Hepatitis E virus (HEV) is a major cause of acute viral hepatitis in lower- and middle-income countries. HEV infection may lead to acute liver failure, chronic liver disease, and high mortality in pregnant women. Antiviral therapy is not a standard treatment for HEV patients. Computational biology tools promise to revolutionize the antiviral drug discovery. Here, we analyzed the transcriptome data of HEV-infected primary human hepatocyte cells through the connectivity map database and applied control theory on the functional network to identify antiviral targets against HEV. The above analyses predicted PKCβ, PKB/AKT, and CK1ε as potential antiviral targets against HEV. The antiviral function of PKB/AKT and CK1ε was experimentally validated by using respective biochemical inhibitors in g3 (genotype 3)-HEV replicon and Huh7 cell-based model of g3 and g1-HEV infection. Furthermore, knockdown of CK1ε showed a similar effect. These data confirmed that CK1ε is an antiviral target for HEV. At present, there are no Food and Drug Administration (FDA)-approved drugs targeting CK1ε. Etravirine is an FDA-approved non-nucleoside reverse transcriptase inhibitor drug, used for the treatment of AIDS patients. An *in silico* study predicted Etravirine to be a potent inhibitor of CK1ε. Our experiments revealed potent antiviral activity of Etravirine against HEV, which was mediated via its ability to inhibit the activity of CK1ε. Taken together, the current study demonstrates that PKB/AKT and CK1ε are bona fide antiviral targets for HEV and paves the way for repurposing Etravirine for the treatment of HEV-infected patients.

**IMPORTANCE:**

Antiviral treatment is not the standard care for acute viral hepatitis E patients. Unbiased identification of antiviral targets or large-scale screening of antiviral compounds against the hepatitis E virus (HEV) has not been reported. Here, a computational biology approach was followed to unbiasedly identify antiviral targets of HEV. Transcriptome data of HEV-infected primary human hepatocyte cells were analyzed to identify modulators of the network and generate directional networks. Network controllability analysis identified PKCβ, PKB/AKT, and CK1ε as potential antiviral targets against HEV. Antiviral function of PKB/AKT and CK1ε was confirmed using cell-based models of genotype 1 (g1)- and g3-HEV infection. Further experiments demonstrated the antiviral activity of Etravirine against HEV, mediated via its ability to inhibit the CK1ε activity. Etravirine is a Food and Drug Administration-approved non-nucleoside reverse transcriptase inhibitor, used for the treatment of AIDS patients. This study reveals the potential of repurposing Etravirine for the treatment of HEV patients and illustrates the importance of computational biology in antiviral drug discovery.

## INTRODUCTION

Hepatitis E Virus (HEV) is a major cause of viral hepatitis globally. It causes acute viral hepatitis in humans, characterized by jaundice, anorexia, nausea, abdominal pain, malaise, fever, and hepatomegaly, which may progress to acute liver failure. It also causes chronic hepatitis, fulminant hepatitis, and extrahepatic manifestations such as Guillain-Barré syndrome and neurological amyotrophy in a subset of patients. The disease worsens during pregnancy, with a 20%–25% mortality rate. Recent reports suggest chronic HEV infection in ~60% of liver transplantation patients (1–4). HEV has eight genotypes and one serotype. HEV genotype 1 (g1) and genotype 2 (g2) infect only humans; genotype 3 (g3) and genotype 4 (g4) infect humans, pigs, wild boars, and deer; genotype 5 (g5) and genotype 6 (g6) infect wild boars, while genotype 7 (g7) and genotype 8 (g8) infect dromedaries and Bactrian camels, respectively (5). Although HEV is a global health issue, it is more common in low- to middle-income countries due to poor sanitation and hygiene. In countries such as Africa, East and South Asia, the disease spreads both as outbreaks and sporadic infection, primarily by g1-HEV and less frequently by g2-HEV infection. Developed countries have only occupational sporadic infection cases, mostly caused by the g3 virus (6). G1-HEV and g2-HEV are transmitted through the fecal-oral route by fecal contamination of drinking water, whereas g3-HEV and g4-HEV are zoonotic and can spread to humans by consumption of uncooked meat from infected animals (7). Vertical transmission of g1-HEV and g2-HEV from mother to child during pregnancy has also been reported (8, 9).

The HEV genome is a positive-sense single-stranded 7.2 kb RNA. 5′-Untranslated region (UTR) of the HEV RNA has a 7-methylguanine-cap, followed by three open reading frames (ORF1, ORF2, and ORF3), and at the 3′-end, there is a UTR and poly-adenine stretch (2). A fourth ORF was identified only in the g1-HEV and named as ORF4 (10). ORF1 encodes the non-structural polyprotein (methyltransferase, protease, helicase, RNA-dependent RNA polymerase, Y-domain, X-domain, and V-domain), involved in viral replication and pathogenesis. The ORF1 polyprotein is translated directly from the HEV genomic RNA, while ORF2 and ORF3 proteins are translated from the sub-genomic RNA. The ORF2 encodes the capsid protein of the HEV. ORF3 is 360 bases long and overlaps with ORF2 in a different reading frame. ORF3 protein interacts with many host proteins, including the tumor susceptibility gene 101 protein (TSG101), a key component of the endosomal sorting complexes required for transport pathway, and this interaction helps in the release of the progeny virion (2). ORF3 protein also modulates multiple host signaling pathways (2). ORF4 encodes the ORF4 protein, which is translated via a cap-independent mechanism, involving an internal ribosome entry site-like element located within the ORF1. ORF4 enhances g1-HEV replication by promoting the assembly of a multiprotein viral replication complex (10).

Antiviral treatment is not the standard care for acute viral hepatitis E patients. Ribavirin monotherapy, pegylated interferon alpha, or a combination of both is considered for viral clearance in chronic hepatitis E, immunocompromised, and organ transplant patients (11–13). However, the side effects of both ribavirin and interferon therapy render the treatment unsuitable for several categories of patients (14–16).

Few studies have been undertaken to identify specific antivirals against HEV. Notably, studies on virus-targeting antiviral screening have identified 3-(4-hydroxyphenyl) propionic acid (viral methyltransferase inhibitor), Favipiravir (viral RNA-dependent RNA polymerase [RdRp] inhibitor) in combination with Sofosbuvir (viral RdRp inhibitor), Methotrexate (viral helicase inhibitor), 2′-C-methylcytidine (viral RdRp inhibitor), and 66E2 (viral RdRp inhibitor) as potential antivirals against HEV (17–21). Studies on host-targeting antivirals have identified compounds such as mycophenolic acid, artesunate, and fenofibrate as potential antivirals against HEV (22–24). Our earlier study identified anti-HEV activity of a cyclic peptide (CP11), which acts by interrupting the interaction between HEV ORF3 and host TSG101 (tumor susceptibility gene 101), leading to the inhibition of progeny virus release (25). Our earlier study also demonstrated the antiviral potential of zinc salts and zinc oxide nanoparticles against HEV (26, 27). Toward identifying potent antiviral targets against HEV in the host, the virus-host protein-protein interaction (PPI) network has been explored by a yeast two-hybrid-based cDNA library screening approach or an immunoprecipitation-mass spectrometry approach using viral proteins as bait (28, 29). Although several potential targets were identified, functional validation of the targets and their small molecule modulators remains unexplored. In summary, although some progress has been made toward identifying specific antivirals against HEV following a target-specific approach or small-scale focused screening approach, unbiased identification of antiviral targets or large-scale screening of antiviral compounds against HEV has not been reported.

A major obstacle in performing high-throughput antiviral screening assays in HEV-infected cells is attributed to the lack of an efficient cell-culture-based infection model of the virus. Alternatively, computational biology methods may be explored to identify antiviral targets and antiviral drugs against HEV using transcriptome and/or proteome profile data of HEV-infected cells and respective controls. One of the approaches for computational identification of antiviral targets is based on analysis of the differential gene expression data set of a diseased/infected/perturbed sample in the connectivity map (CMap) database, construction of a directional PPI network, followed by controllability analysis of the network (30, 31). CMap is a reference drug perturbation data set containing transcriptome profiles of multiple cell lines treated with drugs. Comparison of the transcriptome profile of a disease/infection sample and reference transcriptome profiles available in CMap helps in identifying the inverse drug-disease/infection relation. Control theory analysis of the resulting data helps identify the network’s most fragile nodes. Therefore, a potential target obtained through a combination of CMap and network controllability analysis will not only regulate the network dynamics but also likely induce a transition in the state of the system from “disease” to “healthy” (32).

Todt et al. have reported the host transcriptome profile in g3-HEV-infected primary human hepatocyte (PHH) cells (33). Raw data from the Gene Expression Omnibus (GEO) database were used to select differentially expressed genes (DEGs) at multiple cutoffs and analyze them in the CMap database to identify modulators. Subsequently, DEGs and modulators were used to construct directional PPI networks, followed by network controllability analysis to identify the indispensable modulators. Further analysis identified PRKCB, AKT1, and CSNK1E as potential antiviral targets. Experimental evaluation of HEV inhibitory activity of AKT and casein kinase 1 epsilon (CK1ε) inhibitors revealed anti-HEV activity of respective inhibitor compounds. Further experiments confirmed the antiviral activity of Etravirine against HEV.

Etravirine is a diarylpyrimidine group drug used for the treatment of HIV-1-infected patients. It is a second-generation non-nucleoside reverse transcriptase inhibitor (NNRTI), with the ability to inhibit the HIV-1 strains that are resistant to many other anti-retroviral drugs (34). It has a half-life of around 41 hours, and it gets metabolized in the liver by CYP3A4, CYP2C9, and CYP2C19 enzymes (35). The drug lacks any serious adverse effects except that it leads to the development of rashes in some (36). A drug repurposing study has shown the therapeutic utility of Etravirine against Friedreich’s ataxia, a genetic disorder of progressive loss of coordinated muscle strength (37). In the case of Friedreich’s ataxia, Etravirine acts by increasing the level of frataxin by promoting its translation (37). In a recent study, a search for CK1ε inhibitor from a database of Food and Drug Administration (FDA)-approved drugs identified Etravirine as a potential candidate (38). The CK1 family of protein kinases is important in multiple biological pathways. CK1ε shares around 96% sequence homology with CK1δ. Both kinases are regulated by phosphorylation of their C-terminal tails. In turn, they phosphorylate a set of substrates and control many biological processes, including circadian cycle (PER [period] 2 phosphorylation), cell-cell interaction and cancer (connexin-43 phosphorylation), Cap-dependent translation, regulation of AMPK (AMP-activated protein kinase) pathway and cancer (4EBP1 phosphorylation), and dopamine signaling and drug addiction (dopamine and cAMP-regulated phosphoprotein)−32 phosphorylation (39). CK1ε also phosphorylates TRAF3 (tumor necrosis factor receptor-associated factor 3) and regulates the antiviral innate immune response (40). Given the importance of CK1ε in multiple cellular processes and during viral infection, the potential use of Etravirine as an antiviral drug against HEV is discussed.

## RESULTS

### Transcriptome profile analysis of HEV-infected PHH cells to identify antiviral targets

**Fig 1:**
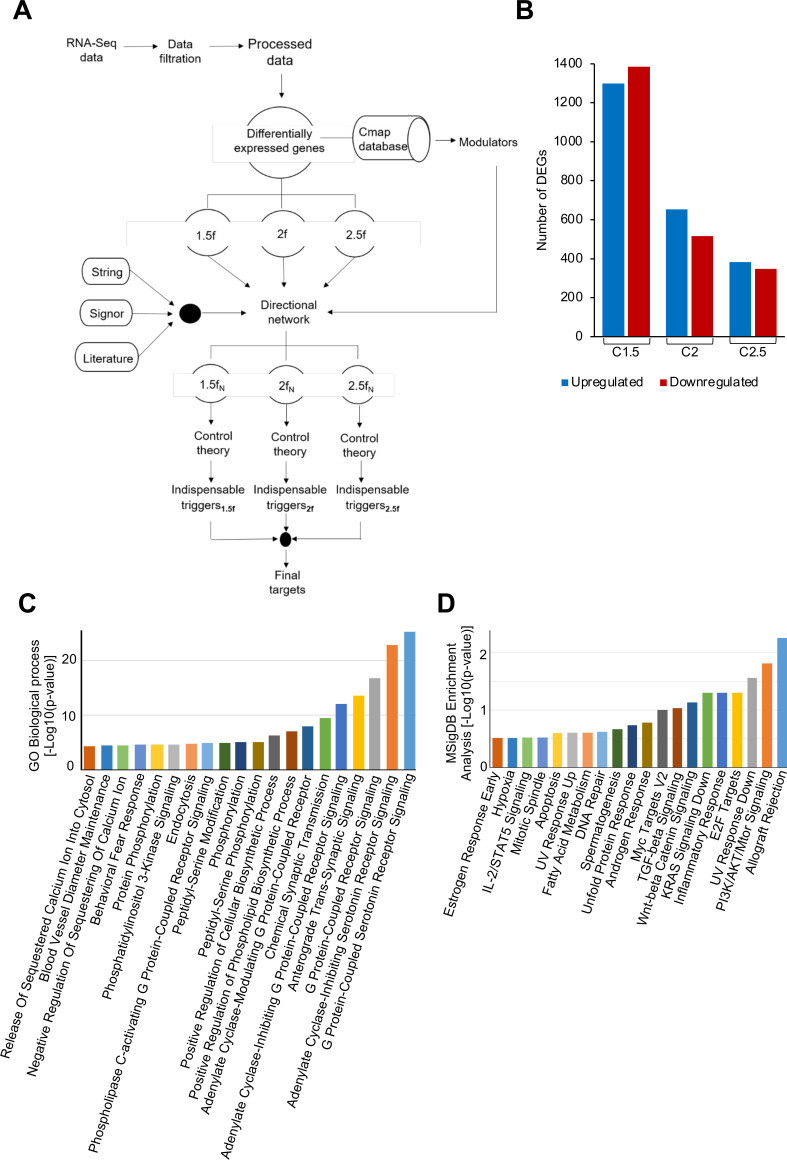
*In silico* analysis of transcriptome of g3-HEV infected PHH cells. (**A**) Schematic of *in silico* analysis. (**B**) Number of DEGs identified at the indicated cutoffs, X-axis: fold change; Y-axis, number of upregulated (blue bar) and downregulated (red bar) genes. (**C**) Top 20 “Gene Ontology (GO) biological processes” associated with the modulators. (**D**) Top 20 “pathways” associated with the modulators obtained by MSigDB enrichment analysis.

Raw data of transcriptome profile of 48 h g3-HEV-infected PHH cells and corresponding uninfected cells were analyzed to identify the DEGs (Fig. 1A). Note that Todt et al. reported a peak level of viral replication to occur at 48 hours post-infection, and the highest number of DEGs was observed at that time point (33). The initial transcriptome data set comprised 21,475 genes, and subsequent data filtration reduced it to 15,094 genes. DEG analysis was performed at 1.5-, 2-, and 2.5-fold cutoffs to ensure that our findings do not unduly rely on a single arbitrary criterion. It also allows detection of a wide range of biologically important variances, from minor (1.5-fold, hereafter denoted as C1.5) to moderate (twofold, hereafter denoted as C2.0) and comparatively severe (2.5-fold, hereafter denoted as C2.5) variations. A total of 1,299, 653, and 382 genes were upregulated, and 1,386, 515, and 348 genes were downregulated at C1.5, C2, and C2.5, respectively (Fig. 1B). Since the CMap portal allows only the top 150 up- and down-regulated genes, CMap analysis for each category resulted in the same set of 36 modulators. The top 20 biological processes and pathways associated with these genes are shown (Fig. 1C and D). Note that the list is dominated by serotonergic receptor encoding genes (11 genes), casein kinase encoding genes (four genes), and adenosine receptor encoding genes (four genes; Table S1).

**Fig 2:**
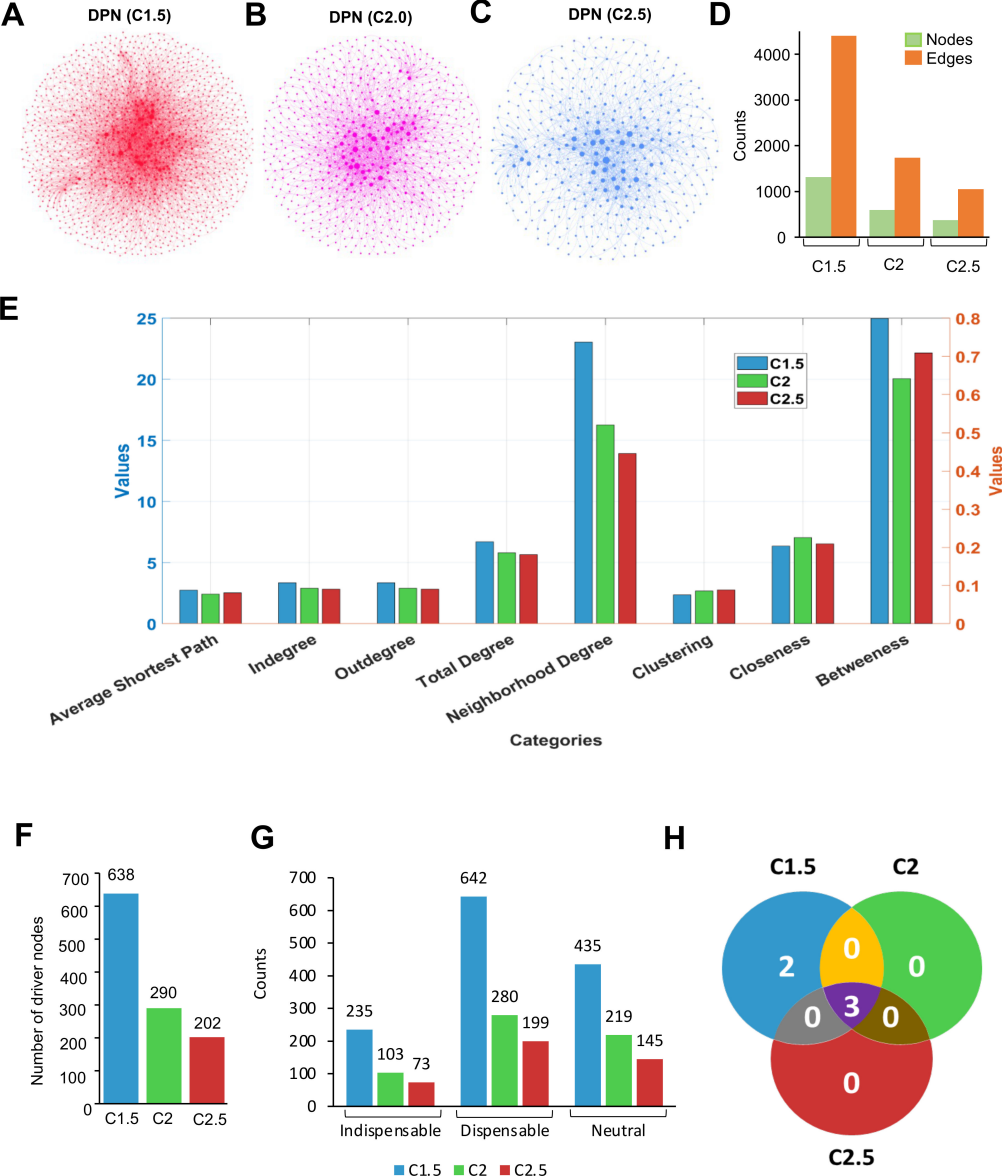
Characteristics of the directional PPI Networks. (**A–C**) DPNs at C1.5, C2, and C2.5 cutoff, nodes are sized according to their degree. (**D**) Nodes and degrees in the C1.5, C2, and C2.5 networks. Green and orange bars represent nodes and edges, respectively. (**E**) Topology analysis of the C1.5, C2, and C2.5 networks. Due to variation in the scale, please refer to the right-hand side y-axis (in red color) for clustering, closeness, and betweenness, and for the rest of the categories, please refer to the left-hand side y-axis (in blue color). (**F**) Driver nodes in the C1.5, C2, and C2.5 networks. (**G**) The number of indispensable, dispensable, and neutral nodes in the C1.5, C2, and C2.5 networks. (**H**) Venn diagram showing the potential targets obtained by analysis of the C1.5, C2, and C2.5 networks.

Next, a directional protein-protein interaction network (DPN) was generated by combining STRING (version 11.0) and Signor (version 2.0), and literature and DEGs and modulators of each category were mapped to it (Fig. 2A through C). A total of 1,314, 600, and 373 nodes, and 4,398, 1,741, and 1,054 edges were detected at C1.5, C2, and C2.5 cutoff, respectively (Fig. 2D). Each displayed one giant component and many small disconnected components (Fig. 2A through C). Analysis of the network properties revealed that C1.5 has the highest betweenness centrality (0.7992), indicating that nodes in C1.5 play a crucial role in connecting other nodes in the network. C2 stands next, followed by C2.5 (Fig. 2E). C2 has the highest closeness centrality (0.2251), suggesting that, on average, nodes in C2 are closer to each other than the other two (Fig. 2E). This easiness in traversing is also reflected in the fact that it has the shortest average path length (2.4269; Fig. 2E). C2.5 has the highest clustering coefficient (0.0883), indicating a higher degree of interconnectedness among neighbors, compared to C1.5 and C2. C1.5 has the highest indegree and outdegree, suggesting that nodes in C1.5 are more connected and receive/send more edges than the other subnetworks. It also has the highest neighborhood degree (23.0393), indicating a higher density of connections in the immediate vicinity of nodes. The values of the centrality measures for each node are provided in Table S2.

To identify the crucial protein(s) governing this network, we applied the network controllability algorithm (31). The number of driver nodes (minimum number of nodes required to control a network) in C1.5, C2, and C2.5 networks was 638, 290, and 202, which correspond to 49.70%, 48.17%, and 47.72%, respectively (Fig. 2F). The large number of driver nodes indicates the sparse nature of these networks. Driver nodes were analyzed using a simple set-theoretic approach to identify indispensable, dispensable, and neutral nodes at each cutoff (Fig. 2G). Five potential targets (PRKCB, IGF1R, DMT1, AKT1, and CSNK1E) were identified at C1.5, of which three (PRKCB, AKT1, and CSNK1E) were retained at C2 and C2.5 (Fig. 2H). As per the above analysis, depletion of PRKCB, AKT1, or CSNK1E or functional inhibition of these proteins using biochemical inhibitors should reverse the HEV infection-induced change in gene expression profile to that of uninfected cells, thereby acting as an antiviral strategy. PRKCB, AKT1, and CSNK1E encode PKCβ, AKT1, and CK1ε proteins, respectively.

### Inhibition of g3- and g1-HEV replication by CK1ε and AKT inhibitors

**Fig 3:**
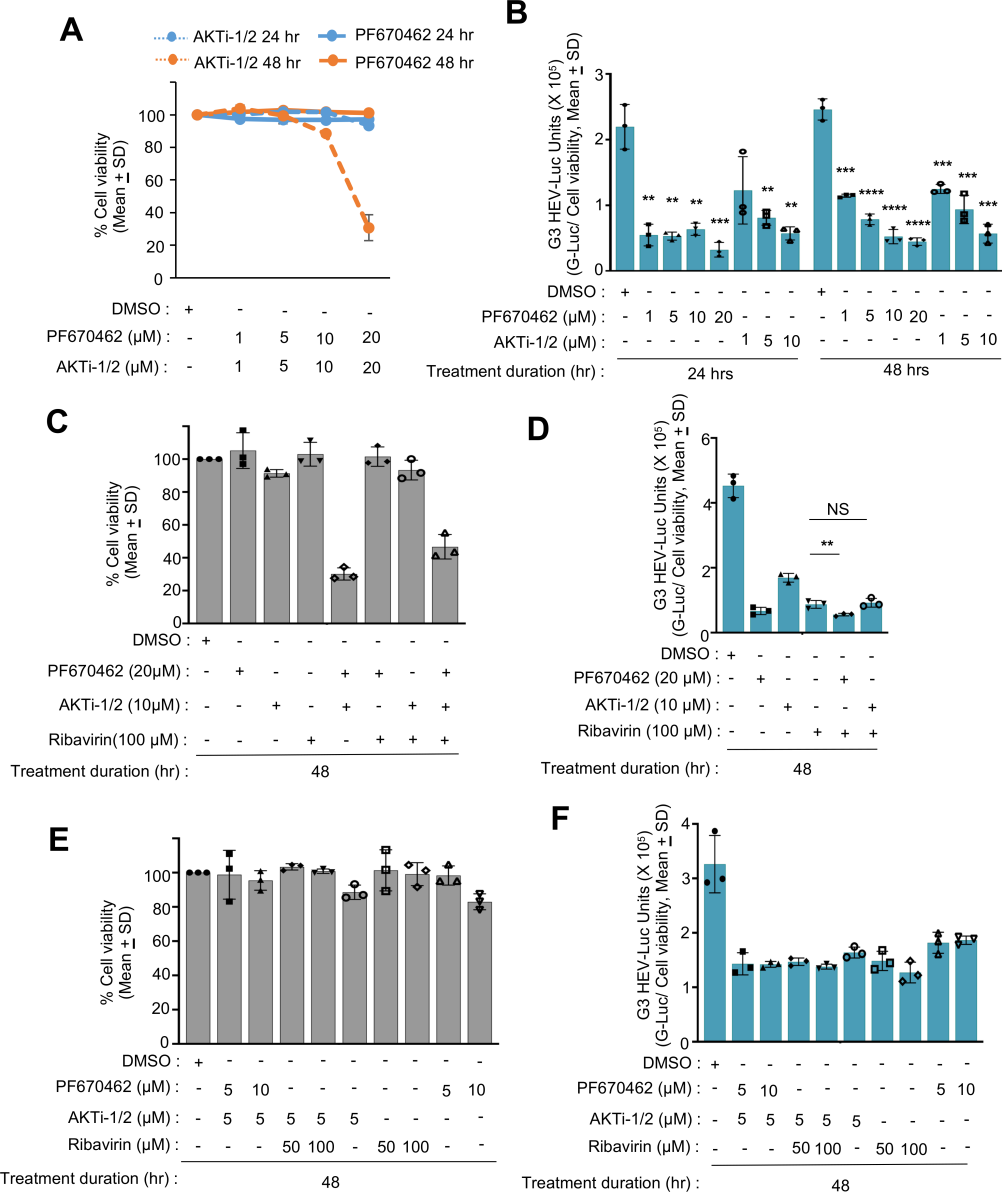
Effect of CK1e and AKT inhibitors on g3-HEV replicon. (**A**) Percent viability (considering Dimethyl Sulfoxide[DMSO]-treated sample value as 100%) in Huh7 cells expressing P6 HEV-Luc- replicon, treated with indicated concentration of PF670462 (CK1e inhibitor) and AKTi-1/2 (AKT inhibitor) for 24 hours and 48 hours. Blue and orange colors represent 24 and 48 hours inhibitor treatment, respectively. Solid and dashed lines represent the cell viability of PF670462- and AKTi-1/2-treated cells, respectively. (**B**) Measurement of *Renilla*-luciferase activity in Huh7 cells expressing in vitro-synthesized capped RNA of a P6 HEV-Luc replicon, treated for 48 hours with different concentrations of PF670462 and AKTi-1/2, as indicated. *Renilla*-luciferase values were normalized to the cell viability values and represented as (mean ± SD) of three independent experiments. (**C**) Percent viability in Huh7 cells expressing P6 HEV-Luc- replicon and treated with the indicated compounds for 48 hours. Viability of the DMSO-treated sample was considered to be 100%, and other values were calculated with reference to that. (**D**) Measurement of *Renilla*-luciferase activity in Huh7 cells mentioned in (**C**). *Renilla*-luciferase values were normalized to the cell viability values and represented as (mean ± SD) of three independent experiments. *P* values were calculated using Student’s *t*-test. (**E**) Percent viability in Huh7 cells expressing P6 HEV-Luc- replicon and treated with the indicated compounds for 48 hours. Cell viability of the DMSO-treated sample was considered to be 100%, and other values were calculated with reference to that. (**F**) Measurement of *Renilla*-luciferase activity in Huh7 cells mentioned in (**E**). *Renilla*-luciferase values were normalized to the cell viability values and represented as (mean ± SD) of three independent experiments. *P* values were calculated using Student’s *t*-test. *, **, *** and **** represent *P* < 0.05, *P* < 0.01, *P* < 0.001, and *P* < 0.0001, respectively.

An earlier study reported a lack of any change in HEV replication in cells lacking PKCβ (41). We evaluated the importance of CK1ε and AKT on HEV replication using PF670462 and AKTi-1/2, biochemical inhibitors of CK1δ/ε and AKT1/2, respectively. Huh7 cells were treated with increasing concentrations of PF670462 and AKTi-1/2 (1 µM, 5 µM, 10 µM, and 20 µM). Twenty-four-hour treatment with up to 20 µM of PF670462 or AKTi-1/2 did not cause any cytotoxicity to Huh7 cells; however, 48-hour treatment (two times treatment at 24-hour interval) with 20 µM AKTi-1/2 caused 50% cell death (Fig. 3A). Therefore, AKTi-1/2 was used at a maximum concentration of 10 µM. Huh7 cells expressing *in vitro* synthesized genomic RNA of g3-HEV-Luc replicon (pSK-P6-HEV-Luc) were treated with increasing concentrations of PF670462 and AKTi-1/2 for 24 hours and 48 hours, followed by measurement of *Gaussia*-Luciferase (G-Luc) activity and cell viability. Treatment of g3-HEV-Luc expressing cells with 20 µM of PF670462 for 48 hours reduced the luciferase activity by approximately 85% in comparison to untreated cells, while treatment of the same cells with 10 µM of AKTi-1/2 for 48 hours reduced luciferase activity by approximately 81% in comparison to untreated cells (Fig. 3B).

Both CK1ε and AKT participate in multiple cellular pathways and act on several targets. It is possible that co-targeting their function might show a cooperative, synergistic, or antagonistic effect on inhibiting HEV replication. Ribavirin is an inosine monophosphate dehydrogenase inhibitor, which is known to inhibit HEV replication and used as an off-label therapeutic in some HEV cases (13). Possible cooperativity or antagonism between PF670462, AKTi-1/2, and ribavirin on HEV replication was evaluated using the g3-HEV-Luc-replicon model. Huh7 cells expressing the g3-HEV-Luc-replicon were treated for 48 hours with 5, 10, and 20 µM PF670462, 5 or 10 µM of AKTi-1/2, and 50 or 100 µM of Ribavirin. Cotreatment with 20 µM PF670462 and 10 µM AKTi-1/2 or 20 µM PF670462, 10 µM AKTi-1/2, and 100 µM ribavirin showed significant cytotoxicity in Huh7 cells (Fig. 3C). No significant change in G-Luc activity was observed in g3-HEV-Luc-expressing Huh7 cells upon cotreatment with 20 µM PF670462 and 100 µM ribavirin or 10 µM AKTi-1/2 and 100 µM ribavirin (Fig. 3D). Furthermore, attempts to optimize the cotreatment dose with varying concentrations of the compounds did not show any cooperative or antagonistic effect (Fig. 3E and F).

**Fig 4:**
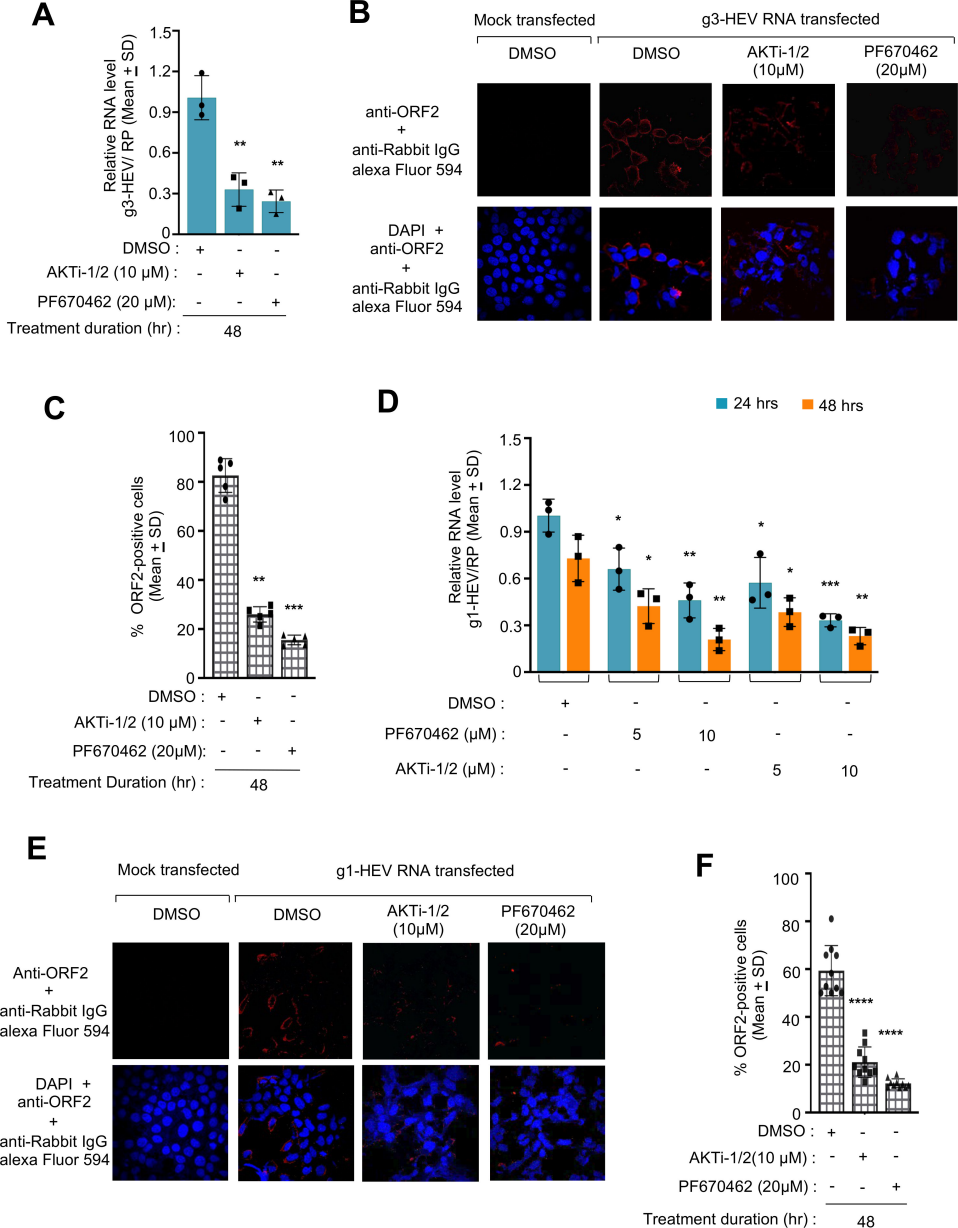
Antiviral activity of CK1ε and AKT inhibitors in g1- and g3-HEV genomic RNA transfected Huh7 cells. (**A**) Real-time quantitative PCR (RT-qPCR) measurement of g3-HEV RNA level in Huh7 cells expressing g3-HEV and treated with PF670462 or AKTi-1/2, as indicated. Relative g3-HEV RNA values were calculated with respect to the value of DMSO-treated samples, normalized with values of Ribonuclease P (RP), and represented as (mean ± SD) of three independent experiments. (**B**) Immunofluorescence image of ORF2 (red, upper panel) or ORF2 (red) and nucleus (blue; lower panel) in Huh7 cells expressing g3-HEV and treated with the indicated inhibitors for 48 hours. (**C**) Quantification of % ORF2-positive cells in five random fields, as represented in panel **B**. Values are (mean ± SD) of five random fields. *P* values were calculated using Student's *t*-test. *P* < 0.05 was considered significant. (**D**) RT-qPCR measurement of g1-HEV RNA in Huh7 cells expressing g1-HEV and treated with PF670462 or AKTi-1/2, as indicated. Relative g1-HEV RNA values were calculated with respect to the value of DMSO-treated samples, normalized with values of RP, and represented as (mean ± SD) of three independent experiments. (**E**) Immunofluorescence image of ORF2 (red, upper panel) or ORF2 (red) and nucleus (blue; lower panel) in Huh7 cells expressing g1-HEV and treated with the indicated inhibitors for 48 hours. (**F**) Quantification of % ORF2-positive cells in 10 random fields, as represented in panel **E**. Values are (mean ± SD) of 10 random fields. *P* values were calculated using Student's *t*-test. *P* < 0.05 was considered significant. *, **, ***, and **** represent *P* < 0.05, *P* < 0.01, *P* < 0.001, and *P* < 0.0001, respectively.

Data obtained in the g3-HEV-Luc replicon model were validated in Huh7 cells expressing a g3-HEV infectious virus. Huh7 cells were electroporated with the *in vitro* synthesized capped genomic RNA of P6-HEV, followed by treatment with 10 µM AKTi-1/2 or 20 µM PF670462, for 48 hours. Both compounds significantly reduced the level of viral RNA, in agreement with results obtained in the g3-HEV-Luc replicon model (Fig. 4A). These results were further confirmed using another commercially available quantitative RT-PCR assay kit, which is approved for the detection and quantification of HEV-specific RNA in patients (RealStar HEV RT-PCR kit 2.0). In this assay, HEV RNA level is quantified as international units per mL (IU/mL) using the World Health Organization International Standard for Hepatitis E Virus RNA Nucleic Acid Amplification Techniques (NAT)-Based Assays. As expected, a similar pattern of g3-HEV inhibition was observed after treatment with the inhibitors (Fig. S1). Immunofluorescent staining of parallelly maintained cells with anti-ORF2 antibody showed a significant reduction in ORF2-positive cells in PF670462- and AKTi-1/2-treated cells (Fig. 4B). Quantification of ORF2-positive cells from five representative fields supports the above observation (Fig. 4C).

Although all HEV genotypes show a single serotype, differences exist between the genotypes in terms of their host range and interaction with host factors (5, 28). As our initial data set was derived from g3-HEV-infected cells, we evaluated the antiviral activity of PF670462 and AKTi-1/2 on g1-HEV, which is predominant in Southeast Asian countries. Huh7 cells were transfected with an *in vitro* synthesized, capped g1-HEV genomic RNA and treated with increasing concentrations of PF670462 (5 µM and 10 µM) and AKTi-1/2 (5 µM and 10 µM), for 24 hours and 48 hours. Real-time quantitative PCR (RT-qPCR) analysis revealed a significant reduction in g1-HEV RNA level in 5 µM and 10 µM PF670462-treated cells at both 24 hours and 48 hours treatment period, later being more efficient (Fig. 4D). A similar pattern was seen in AKTi-1/2 treated cells (Fig. 4D; Fig. S2). Next, an immunofluorescence assay was performed after 48 hours treatment of g1-HEV-expressing Huh7 cells with 20 µM PF670462 or 10 µM AKTi-1/2. As expected, there was a significant reduction in ORF2-positive cells upon treatment with PF670462 or AKTi-1/2 (Fig. 4E and F).

**Fig 5:**
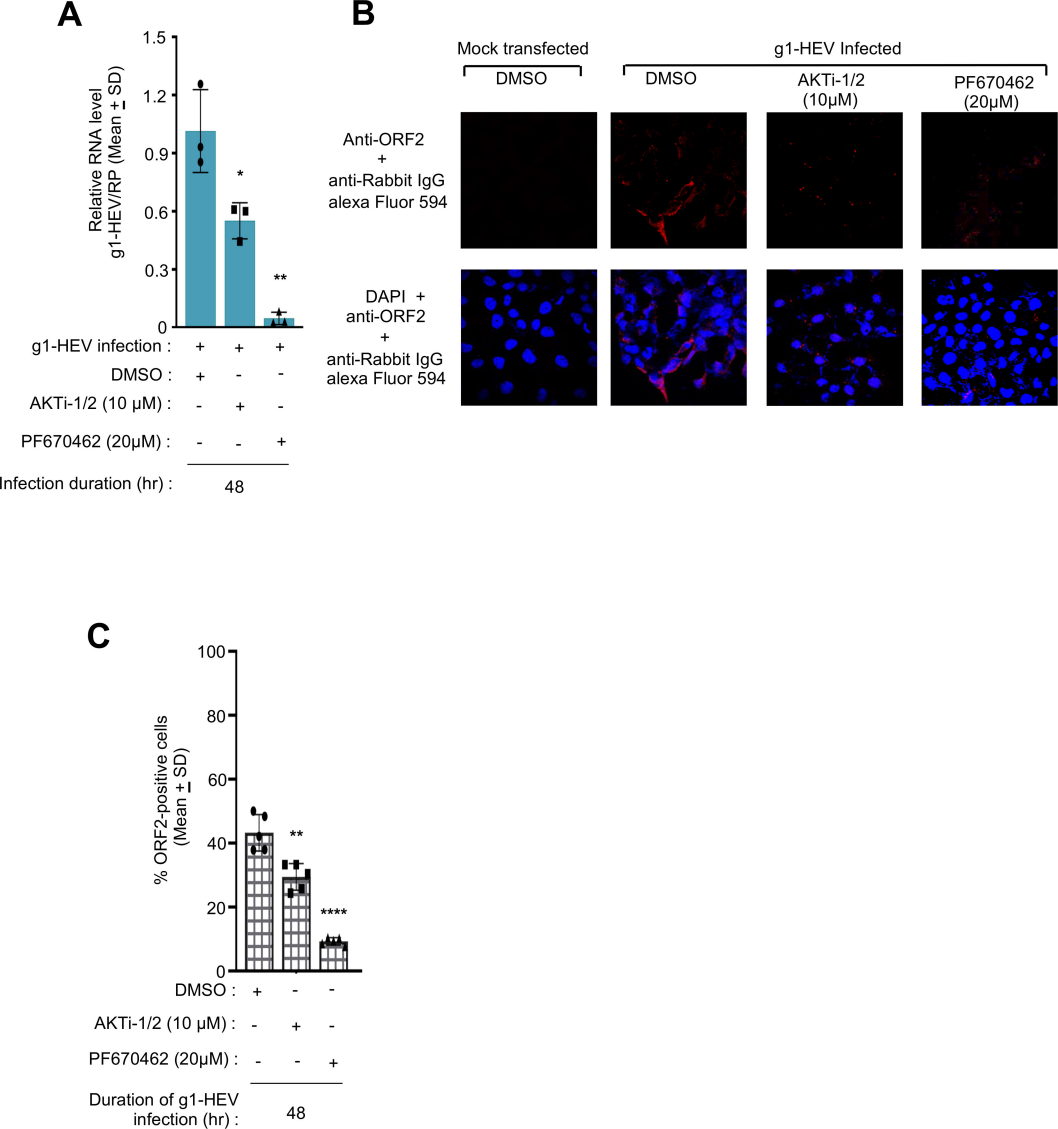
Antiviral activity of CK1ε and AKT inhibitors in g1-HEV clinical isolate-infected Huh7 cells. (**A**) RT-qPCR measurement of g1-HEV RNA in g1-HEV clinical isolate-infected Huh7 cells and treated with PF670462 or AKTi-1/2, as indicated. Relative g1-HEV RNA values were calculated with respect to the value of DMSO-treated samples, normalized with values of RP, and represented as (mean ± SD) of three independent experiments. (**B**) Immunofluorescence image of ORF2 (red, upper panel) or ORF2 (red) and nucleus (blue; lower panel) in g1-HEV clinical isolate-infected Huh7 cells and treated with the indicated inhibitors for 48 hours. (**C**) Quantification of % ORF2-positive cells in five random fields, as represented in (**A**). Values are (mean ± SD) of five random fields. *P* values were calculated using Student's *t*-test. *P* < 0.05 was considered significant. *, **, ***, and **** represent *P* < 0.05, *P* < 0.01, *P* < 0.001, and *P* < 0.0001, respectively.

Next, the antiviral activity of PF670462 and AKTi-1/2 was tested on a g1-HEV clinical isolate. The virus was purified from the fecal sample of an acute HEV patient, followed by infection of Huh7 cells and measurement of viral replication in the presence and absence of 20 µM PF670462 or 10 µM AKTi-1/2. Viral replication was measured by RT-qPCR and immunofluorescence assay. In agreement with the earlier results, a significant reduction in g1-HEV RNA and protein levels was observed in infected cells upon treatment with PF670462 or AKTi-1/2 (Fig. 5A through C; Fig. S3).

### CK1ε is essential for efficient replication of HEV

**Fig 6:**
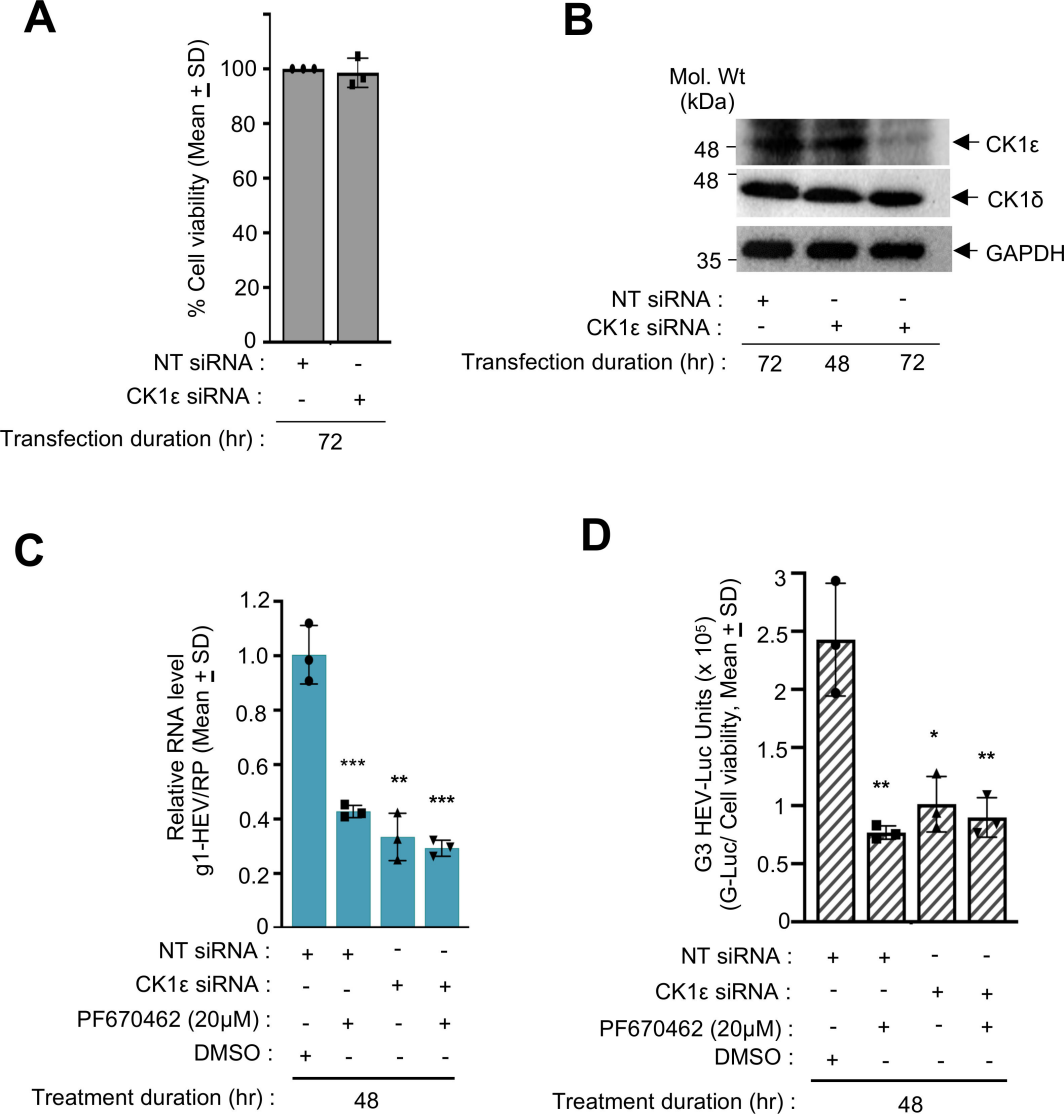
Knockdown of CK1ε inhibits g1- and g3-HEV replication. (**A**) Percent viability in Huh7 cells transfected for 72 hours with NT-siRNA (non-targeting siRNA) or CK1ε siRNA, as indicated. The values of NT-siRNA-transfected samples were considered as 100%. (**B**) Western blot analysis of CK1ε (upper panel), CK1δ (middle panel), and GAPDH (lower panel) protein levels in Huh7 cells, transfected with NT-siRNA or CK1ε siRNA for 48 or 72 hours, as indicated. (**C**) RT-qPCR measurement of g1-HEV RNA in Huh7 cells expressing g1-HEV, transfected with NT-siRNA or CK1ε siRNA for 72 hours and treated with PF670462 or DMSO for 48 hours, as indicated. Relative g1-HEV RNA values were calculated with respect to the value of DMSO-treated samples, normalized with values of RP, and represented as (mean ± SD) of three independent experiments. (**D**) Measurement of *Renilla*-luciferase activity in Huh7 cells expressing g3-HEV-Luc-replicon and transfected with NT-siRNA or CK1ε siRNA for 72 hours and treated with PF670462 or DMSO for 48 hours, as indicated. *Renilla*-luciferase values were normalized to the cell viability values and represented as mean ± SD of three independent experiments. *, **, ***, and **** represent *P* < 0.05, *P* < 0.01, *P* < 0.001, and *P* < 0.0001, respectively.

In order to confirm the role of CK1ε in mediating HEV replication, Small Interfering RNA (siRNA) against CK1ε was used to deplete the corresponding protein from Huh7 cells, followed by quantification of HEV replication. Huh7 cells were transfected with 25 nM of CK1ε siRNA and NT-siRNA (non-targeted siRNA). Seventy-two hours post-transfection, the viability of the cells was measured by 3-(4,5-dimethylthiazol-2-yl)-5-(3-carboxymethoxyphenyl)-2-(4-sulfophenyl)-2H-tetrazolium (MTS) assay. No difference was observed between NT-siRNA and CK1ε siRNA-treated cells, ruling out any cytotoxicity of the siRNAs on Huh7 cells (Fig. 6A). Western blot analysis of parallelly maintained cells using anti-CK1ε antibody showed a marked reduction of CK1ε in corresponding siRNA-treated cells (Fig. 6B, upper panel). However, there was no change in CK1δ (CK1 Delta) protein level, confirming the specificity of the siRNA against CK1ε (Fig. 6B, middle panel). The level of GAPDH was measured as a control to ensure equal loading of protein (Fig. 6B, lower panel). Next, g1-HEV-expressing Huh7 cells were treated with the CK1ε siRNA and/or treated with PF670462, followed by quantification of viral RNA level by RT-qPCR. Treatment with CK1ε siRNA or PF670462 inhibited g1-HEV level by ~70% (Fig. 6C). However, cotreatment with siRNA and PF670462 showed no further inhibition (Fig. 6C; Fig. S4). A similar trend was observed in g3-HEV-Luc expressing cells (Fig. 6D).

### Antiviral activity of Etravirine against HEV

A literature search was performed to identify FDA (USA)-approved drugs that inhibit the activity of CK1ε. Recently, CK1ε inhibitor Umbralisib was granted FDA approval for the treatment of marginal zone lymphoma and follicular lymphoma (42). However, it was withdrawn following safety issues (43). In another drug repurposing study, FDA-approved drug Etravirine was identified as a potential inhibitor of CK1ε (38). Etravirine was originally identified as a non-nucleoside reverse transcriptase inhibitor, used for the treatment of HIV-1-infected patients (34, 35). We evaluated the antiviral activity of Etravirine against HEV in g3- and g1-HEV genomic RNA transfected and g1-HEV clinical isolate-infected Huh7 cells.

**Fig 7:**
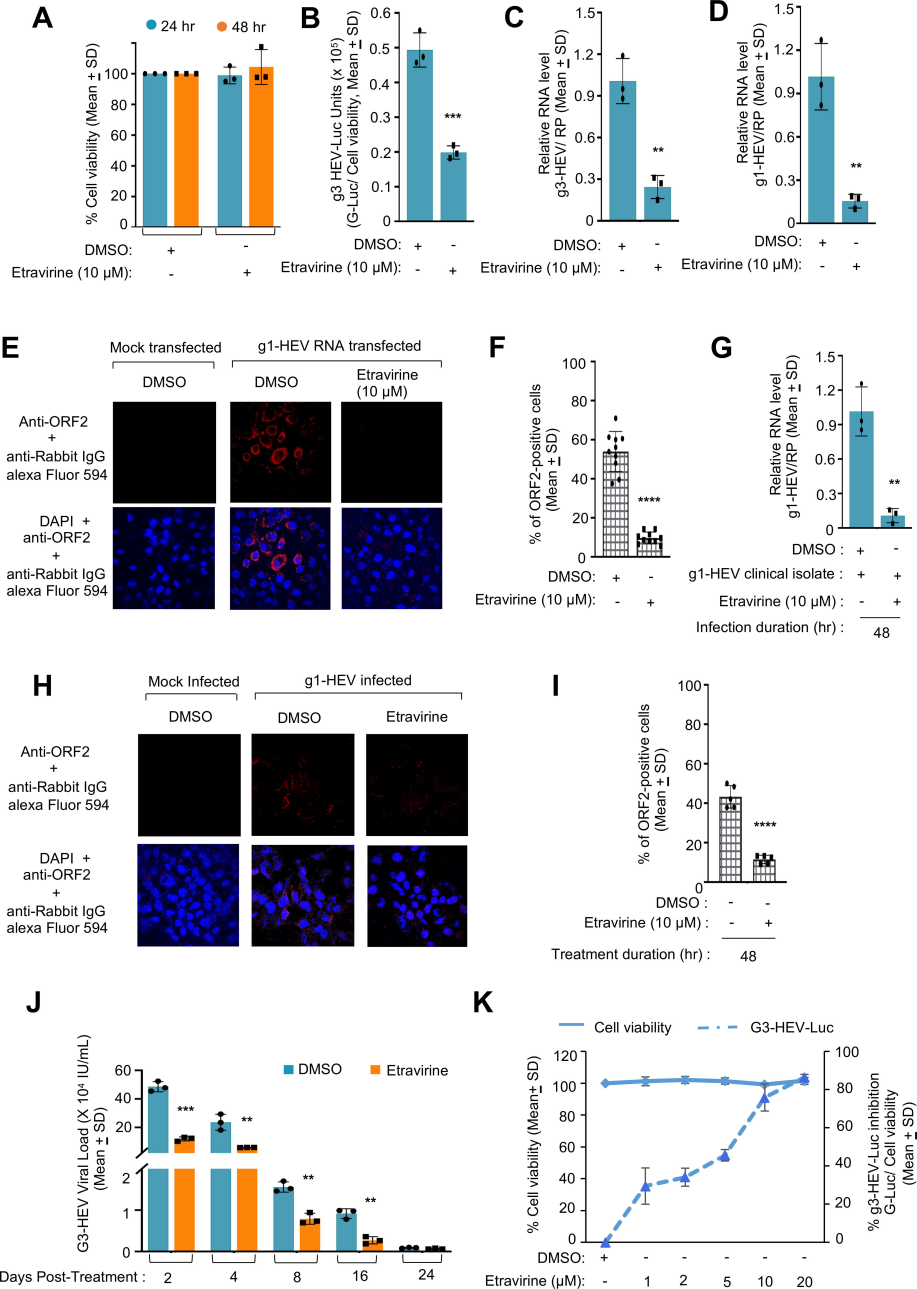
Antiviral activity of Etravirine against g1- and g3-HEV. (**A**) Percent viability of Huh7 cells upon treatment with 10 µM Etravirine or DMSO for 24 or 48 hours, as indicated. The values of DMSO-treated samples were considered as 100%. (**B**) Measurement of *Renilla*-luciferase activity in Huh7 cells expressing g3-HEV-Luc-replicon and treated with 10 µM Etravirine or DMSO for 24 or 48 hours, as indicated. *Renilla*-luciferase values were normalized to the cell viability values and represented as (mean ± SD) of three independent experiments. (**C**) RT-qPCR measurement of g3-HEV RNA level in Huh7 cells expressing g3-HEV and treated with 10 µM Etravirine for 48 hours, as indicated. Relative g3-HEV RNA values were calculated with respect to the value of DMSO-treated samples, normalized with values of RP, and represented as (mean ± SD) of three independent experiments. *P* values were calculated using the Student *t*-test. *P* < 0.05 was considered significant. (**D**) RT-qPCR measurement of g1-HEV RNA level in Huh7 cells expressing g1-HEV and treated with 10 µM Etravirine for 48 hours, as indicated. Relative g1-HEV RNA values were calculated with respect to the value of DMSO-treated samples, normalized with values of RP, and represented as (mean ± SD) of three independent experiments. *P* values were calculated using Student’s *t*-test. *P* < 0.05 was considered significant. (**E**) Immunofluorescence image of ORF2 (red, upper panel) or ORF2 (red) and nucleus (blue; lower panel) in Huh7 cells expressing g1-HEV and treated with 10 µM Etravirine for 48 hours. (**F**) Quantification of % ORF2-positive cells in five random fields, as represented in panel **E**. Values are (mean ± SD) of five random fields. *P* values were calculated using Student's *t*-test. *P* < 0.05 was considered significant. (**G**) RT-qPCR measurement of g1-HEV RNA level in g1-HEV clinical isolate-infected Huh7 cells and treated with 10 µM Etravirine for 48 hours, as indicated. Relative g1-HEV RNA values were calculated with respect to the value of DMSO-treated samples, normalized with values of RP, and represented as (mean ± SD) of three independent experiments. *P* values were calculated using Student’s *t*-test. *P* < 0.05 was considered significant. (**H**) Immunofluorescence image of ORF2 (red, upper panel) or ORF2 (red) and nucleus (blue; lower panel) in g1-HEV clinical isolate-infected Huh7 cells and treated with 10 µM Etravirine for 48 hours. (**I**) Quantification of % ORF2-positive cells in five random fields, as represented in (**H**). Values are (mean ± SD) of five random fields. *P* values were calculated using Student's *t*-test. *P* < 0.05 was considered significant. (**J**) RT-qPCR measurement of g3-HEV RNA kinetics in g3-HEV electroporated Huh7 cells treated with 10 µM of Etravirine at the indicated time points. The g3-HEV RNA values were calculated with respect to that of the standard and represented as (mean ± SD) of three independent experiments. (**K**) Three-axis graph showing percent viability (Y axis, left side scale) and percent inhibition of *Renilla*-luciferase activity (Y axis, right side scale) in Huh7 cells treated with 10 µM Etravirine or DMSO for 24 hours, as indicated. The values of DMSO-treated samples were considered as 100%. *Renilla*-luciferase values were normalized to the cell viability values. Percent inhibition was calculated with reference to the values obtained in DMSO-treated samples. Values are (mean ± SD) of three independent experiments. Solid line represents percent cell viability, and dashed line represents percent inhibition of g3-HEV-Luc. *, **, ***, and **** represent *P* < 0.05, *P* < 0.01, *P* < 0.001, and *P* < 0.0001, respectively.

The possible cytotoxic effect of Etravirine on Huh7 cells was checked by measuring the viability of Etravirine-treated Huh7 cells at 24 hours and 48 hours post-treatment with the drug. Forty-eight-hour treatment with 10 µM Etravirine did not show any cytotoxicity on Huh7 cells (Fig. 7A). Next, the effect of etravirine treatment on g3-HEV replication was measured by using the g3-HEV-Luc-replicon model. Treatment with 10 µM Etravirine for 48 hours inhibited G-Luc activity by ~60% (Fig. 7B). RT-qPCR analysis revealed a similar antiviral effect of Etravirine in infectious g3-HEV and infectious g1-HEV genomic RNA-expressing Huh7 cells (Fig. 7C and D; Fig. S5A and B). In agreement with the RT-qPCR results, immunofluorescence analysis of g1-HEV RNA transfected Huh7 cells showed a significant reduction in ORF2-positive cells upon 48 hours of treatment with 10 µM Etravirine (Fig. 7E and F). Next, Huh7 cells were infected with the g1-HEV clinical isolate and treated with 10 µM Etravirine for 48 hours, followed by measurement of viral replication. As expected, both viral RNA and ORF2 protein levels were significantly lower in the presence of Etravirine, further supporting the antiviral activity of the compound (Fig. 7G through I; Fig. S5C). To evaluate the long-term effect of Etravirine on HEV, infectious g3-HEV RNA-expressing Huh7 cells were maintained for a longer period in the presence and absence of Etravirine, treated every alternate day. A significant level of HEV RNA could be detected up to 16 days post-electroporation (Fig. 7J, DMSO-treated samples). As expected, there was a significant reduction in HEV RNA level in Etravirine-treated samples (Fig. 7J). Next, the IC_50_ (50% inhibitory concentration) of Etravirine against g3-HEV was evaluated by measuring cell viability and G-Luc activity in the presence of increasing doses of the compound, which revealed its IC_50_ to be ~5 µM (Fig. 7K). Taken together, these results demonstrate the antiviral activity of Etravirine against g1- and g3-HEV.

## DISCUSSION

In the current study, we followed the computational biology approach to discover novel antiviral targets against HEV and characterized the antiviral potential of AKT/PKB and CK1ε inhibitors. Transcriptome data of HEV-infected PHH cells were used as source data for our computational analysis. Compared to transformed cell lines, PHH cells are closer to normal hepatocytes; therefore, the results obtained using the PHH-based HEV infection model are likely to be relevant for downstream applications. Analysis was started with the identification of DEGs at multiple cutoffs, which likely contributes to the overall validity and comprehensiveness of the study’s conclusions. Next, gene reversibility analysis was performed using the CMap database to determine which genes can reverse the differentially expressed gene profile. Next, network controllability analysis was applied to identify the indispensable modulators. Network controllability analysis is one of the most promising algorithms that stands over many of its counterparts (30, 31). It refers to the study and understanding of how to control or influence the behavior of a complex network by manipulating a small set of strategically chosen nodes. By amalgamating network controllability and disease reversibility, we dissected the regulatory architectures governing gene expression changes following virus propagation and identified the key control sets that shape the emergent behaviors of the disease systems. To improve the robustness of the analysis, we opted for a multiple cutoff approach with three different fold changes of the differentially expressed genes. This approach captures the inherent variability of biological networks that define the regulatory landscape, resulting in a more thorough and nuanced knowledge of the underlying system. By capturing modulators that retain indispensability across a broad range of perturbations, this approach likely increases the potency of the development of broad-spectrum therapeutic strategies with far-reaching clinical implications.

An antiviral against HEV is necessary, and attempts have been made in the past in that direction (17–27). However, at present, no prescription drug is available against HEV. Literature search did not show any report on anti-HEV activity of PKCβ, AKT1, and CK1ε inhibitors. However, it has been reported that knockdown of PKCβ does not alter HEV replication (41). Therefore, we did not pursue further studies on PKCβ. It is reported that phosphatidyl inositol three kinase (PI3K) inhibitor LY294002 and mechanistic target of rapamycin (mTOR) inhibitor rapamycin facilitate HEV replication (44). A similar profile was obtained upon LY294002 treatment of g3-HEV replicon expressing Huh7 cells in our laboratory (data not shown). It is also reported that Phospho-AKT (S473) level is higher in Huh7 cells lacking mTOR as well as cells expressing g3-HEV (44). Upon activation, PI3K stimulates the production of phosphatidylinositol (3,4,5)-trisphosphate, which binds to the pleckstrin homology (PH)-domain of AKT and recruits it to the plasma membrane, where 3-phosphoinositide-dependent kinase 1 phosphorylates Thr308 residue of its kinase domain. Complete activation of AKT requires phosphorylation at its Ser473 residue by any of the following kinases, including PDK-1, integrin-linked kinase, DNA-dependent protein kinase, or AKT itself. Given the complexity of PI3K-AKT signaling, available data are insufficient to predict the proviral or antiviral role of AKT in HEV-infected cells.

Activated AKT phosphorylates a variety of downstream proteins such as mTORC1, cell cycle-dependent kinase inhibitor p21 and p27, FOXO1, WEE1, GSK3β, and IKK (45). Phosphorylation by AKT leads to degradation of FOXO as well as cell cycle inhibitors p21 and p27 (46–48). AKT-mediated phosphorylation also inactivates GSK3, which is a negative regulator of cell cycle progression (49). AKT also suppresses TSC1/2 formation and activates Rheb, an activator of mTORC1, which further phosphorylates ribosomal protein S6 kinase (S6K) and 4E-BP1. Phosphorylation of 4E-BP1 releases eIF4E. Activated S6K and eIF4E promote protein translation and cell proliferation (45).

We investigated the crosstalk between AKT, CK1ε, and HEV using respective inhibitors. Both Akti-1/2 and PF670462 (well-characterized inhibitors of AKT and CK1δ/ε, respectively) inhibited HEV replication in Huh7 cells. Akti-1/2 is a cell-permeable, reversible, and selective inhibitor of AKT1/AKT2 activity. Its IC_50_ for AKT1 and AKT2 is 58 nM and 210 nM, respectively. It binds to the PH domain of AKT and blocks basal and stimulated phosphorylation/activation of AKT1/AKT2, both in cultured cells *in vitro* and in mice *in vivo* (50). Recently, another AKT inhibitor, Capivasertib, has been approved for the treatment of adult patients with hormone receptor-positive, human epidermal growth factor receptor 2-negative localized or metastatic breast cancer (51). Its antiviral potential against HEV should be evaluated.

CK1ε is a serine-threonine kinase of the CK1 family. The CK1 family has seven members (α, β, γ1, γ2, γ3, δ, and ε), in which ck1ε shares the highest similarity with ck1δ in the C-terminal domain. CK1ε plays a pivotal role in several signaling pathways that control the circadian clock, cell proliferation, vesicle trafficking, DNA replication, and repair (52, 53). Notably, it controls the circadian cycle by phosphorylating cytoplasmic PER, leading to its degradation; phosphorylates disheveled 2 (Dvl2), a key component of the Wnt/β-catenin pathway, phosphorylates and inactivates 4EBP1, promoting translation initiation (54). Dvl2 phosphorylation by Ck1ε is dependent on its association with DDX3 (considered as a regulatory subunit of Ck1ε) (55). Ck1ε has also been reported to bind to ATP-dependent RNA helicase DDX3X allosterically, activating Wnt signaling (56). On the other hand, AMPK phosphorylates Ser389 of Ck1ε and upregulates its kinase activity (57).

Antiviral activity of two Ck1ε inhibitors was evaluated in this study. PF670462 is a selective inhibitor of CK1δ and CK1ε kinases, with IC_50_ of 7.7 nm and 14 nm, respectively. However, it is not FDA approved. Umbralisib, a dual kinase inhibitor of PI3K-δ and CK1ε, was granted FDA approval in 2021 for the treatment of marginal zone lymphoma and follicular lymphoma (42). However, the approval was withdrawn in 2022 due to safety concerns (43). Etravirine is a second-generation NNRTI. It is FDA approved for the treatment of HIV-1-infected patients. EC_50_ of Etravirine against wild-type HIV-1 strain is 4 ng/mL in acutely infected T-cell lines, and the half-life of Etravirine is ~41 hours. It is safe for clinical use, with minor rash being the side effect in some cases (36). Both inhibitors were equally effective in inhibiting the replication of g1- and g3-HEV. The exact molecular mechanism by which Etravirine and other Ck1ε inhibitors block HEV replication remains to be investigated. We investigated the possibility of HEV RdRp being a substrate of Ck1ε. Phospho-proteomics analysis of mammalian cell-purified RdRp did not reveal any Ck1ε-phosphorylated residue, thereby ruling out a direct effect of Ck1ε on HEV RdRp (unpublished data). Nonetheless, Ck1ε might be modulating HEV replication by acting on host pathways. For example, DDX3 is known to be a pro-viral factor for HEV replication (58). As mentioned above, DDX3 is a regulatory subunit of Ck1ε. Thus, the effect of DDX3 and Ck1ε on HEV replication may be linked. Moreover, enhanced translation due to CK1ε-mediated phosphorylation of 4E-BP1 might facilitate the synthesis of viral proteins, which in turn enhances viral replication. Etravirine belongs to the group of diarylpyrimidine derivatives. Recently, additional diarylpyrimidine derivatives were reported to possess better antiviral activity on HIV-1 and lower toxicity, compared to Etravirine (59). Anti-HEV activity of such molecules should be evaluated.

In summary, following computational analysis-mediated identification of antiviral targets against HEV and experimental validation of the targets in a mammalian cell culture-based model of HEV replication, this study demonstrates the potent antiviral activity of Etravirine against HEV. Etravirine inhibits both g1- and g3-HEV, which are the most common genotypes to infect humans. Given that etravirine is already used in the treatment of AIDS patients without any known side effects, it is a promising candidate for further evaluation as an anti-HEV drug in humans. Our study also paves the way for designing novel antiviral strategies against HEV by targeting host Ck1ε and AKT/PKB.

## MATERIALS AND METHODS

### Computational analysis

The RNA-seq data reported by Todt et al. were downloaded from the GEO database (accession no. GSE135619) (33). Raw data of control and g3-HEV-infected groups were filtered to remove genes with null Reads Per Kilobase Million (RPKM) in both categories. DEGs were identified at threefold change cutoffs (1.5, 2, and 2.5; Fig. 1A). CMap and network controllability analyses were done as described (32). Briefly, the DEGs from different cutoffs were independently analyzed in the CMap database, and genes that showed a connectivity score cutoff of −90 or less were selected (60). These genes are hereafter referred to as modulators. A DPN was generated by combining STRING (version 11.0), Signor (version 2.0), and literature (61). DEGs and modulators of each category were combined and mapped to the DPN to construct three directional subnetworks. Next, structural network controllability analysis was performed following the algorithm published by Liu and co-workers to identify the indispensable nodes in the network (31). Indispensable modulators were identified by a simple set-theoretic approach, using the following equation:


Indispensable Modulators=Modulators∩indispensable nodes.

### Plasmids and reagents

The pSK-HEV-2 plasmid (GenBank accession number AF444002.1), pSK P6-Luc (GenBank accession no. JQ679013.1), and pSK-P6 HEV plasmid (GenBank accession number JQ679013), containing the cDNA of g1-HEV, g3-HEV replicon, and g3-HEV, respectively, have been described (21). These plasmids were generously provided by Emerson (62, 63). The anti-CK1ε antibody (Catalog No. sc-365259) was from Santa Cruz Biotechnology (Texas, USA). The anti-CK1d antibody (Catalog No. 12417) was from Cell Signaling Technology (Massachusetts, USA). The anti-GAPDH antibody (Catalog no. AC001) was from Abclonal (Woburn, MA, USA). The goat anti-Mouse Immunoglobulin G-Horseradish Peroxidase (IgG-HRP) (Catalog No. 1030-05) was from Southern Biotech (Alabama, USA). mMessage mMachine T7 transcription kit (Catalog No. AM 1344), lipofectamine 3000 (Catalog No. L3000075), and goat anti-Rabbit IgG-HRP (Catalog No. 31460) were from Thermo Fisher Scientific (Massachusetts, USA). PF670462 (Catalog No. S6734) and Etravirine (Catalog No. S3080) were from Selleckchem (Texas, USA). AKTi-1/2 (Catalog No. 124018) was from Sigma (Missouri, USA). DharmaFECT transfection reagent (Catalog No. T-2005) was from Dharmacon (CO, USA). CellTiter 96 aqueous one solution cell proliferation assay kit was from Promega (Madison, USA). RealStar HEV RT-PCR Kit 2.0 was from Altona Diagnostics (Hamburg, Germany).

### Mammalian cell culture, siRNA transfection, and cell viability assay

Human hepatoma (Huh7) cells were maintained in Dulbecco’s Modified Eagle Medium containing 10% fetal bovine serum, penicillin, and streptomycin in a humidified incubator with 5% CO_2_ at 37°C, as described (26). For siRNA-mediated gene silencing, Huh7 cells were transfected with 25 nM of NT-siRNA or CK1e siRNA (5′ GCCGUCGAGAUGACCUGGA 3′), which was designed using the GenScript siRNA target finder tool and synthesized at Eurogentec (Liege, Belgium), using DharmaFECT transfection reagent according to the manufacturer’s recommendations.

Stock solution of PF670462, AKTi-1/2, Ribavirin, and Etravirine was prepared in DMSO and diluted in culture medium during treatment of the cells. Cell viability was measured using a commercially available kit (CellTiter 96 Aqueous One Solution Cell Proliferation Assay [Promega, Madison, USA]), which utilizes a tetrazolium salt-based colorimetric assay, following the manufacturer’s instructions.

### RNA isolation, RT-qPCR, western blot, and immunofluorescence assay

Total RNA was isolated using TRI reagent (MRC, Massachusetts, USA). Real Time-Quantitative Polymerase Chain Reaction (RT-qPCR) was done using SOLIScript 1-step probe kit according to the manufacturer’s recommendation (Solis BioDyne, Tartu, Estonia). Following primers were used: g1-HEV FP: 5′TATACTCGAGGGTGCCGATCGGTCCC3′; g1-HEV RP:5′ TATACCATGGCATCTGGCAGCAAGCTCAG3′; g1-HEV Probe: 5′[FAM] TTGACGCCTGGGAGCGGAATCACC [BHQ1]3′; Ribonuclease P (RP) FP: 5′AGATTTGGACCTGCGAGCG3′; RP RP: 5′GAGCGGCTGTCTCCACAAGT3′; RP Probe: 5′[FAM]TTCTGACCTGAAGGCTCTGCGCG[BHQ1]3′; g3-HEV FP: 5′TTACGGTTCACGAAGCTCAG3′; g3-HEV RP: 5′GGCGGGTAAGTGCAACTAT3′; g3-HEV probe: 5′[FAM]TGAGACCACAGTTATAGCCACGGC[BHQ1]3′. Relative quantification of HEV RNA level was done using the 2−ΔΔCT method. Values were calculated relative to those obtained for the DMSO-treated samples and represented as mean (+SD) of triplicate samples. RP was used as a control gene for normalization of the data. Western blot and immunofluorescence assays were done as described (26).

For measurement of HEV RNA in IU/mL, RT-qPCR was performed using RealStar HEV RT-PCR Kit 2.0, following the manufacturer’s guidelines (Altona Diagnostics, Hamburg, Germany). The assay includes a heterologous amplification system (Internal Control) to identify possible RT-PCR inhibition and to confirm the integrity of the reagents of the kit. The assay includes quantification standards that contain standardized concentrations of HEV-specific RNA. These quantification standards were calibrated against the 1st World Health Organization International Standard for Hepatitis E Virus RNA Nucleic Acid Amplification Techniques (NAT)-Based Assays (PEI code 6329/10). The quantification standards were used individually as positive controls and to generate a standard curve, allowing determination of the concentration of HEV-specific RNA in a sample. To determine the viral load of the original sample, the following formula was to be applied: viral load (sample) [IU/mL]= {volume of (eluate) [μL] × viral load (eluate) [IU/μL]}/sample input [mL], as per the manufacturer’s suggestion. The HEV RNA level was plotted as mean + SD of triplicate samples.

### G1-HEV, g3-HEV, and g3-HEV-Luc-replicon RNA synthesis and measurement of viral replication in Huh7 cells

pSK-HEV-2 plasmid containing the cDNA of g1-HEV genome was linearized with Bgl*II*. pSK-P6 HEV and pSK-P6 HEV-Luc plasmids containing cDNAs of wild-type g3-HEV and g3-HEV-Luc-replicon were linearized with Mlu*I*. Linearized plasmids were purified and used for *in vitro* transcription using the mMessage mMachine T7 transcription kit, following the manufacturer’s instructions. The size and integrity of the *in vitro* transcribed RNAs were monitored by formaldehyde agarose gel electrophoresis. g1-HEV RNA was transfected into Huh7 cells using lipofectamine 3,000, as described (26). g3-HEV-Luc and g3-HEV RNA were electroporated into Huh7 cells, as described with the following conditions (4 mm cuvette, 200 V, 950 µF, and infinite resistance) (26). Seventy-two hours post-transfection or post-electroporation, g1- and g3-HEV expressing cells were treated with the compounds for 24 and 48 hours, as indicated. Viral replication was measured by TaqMan-based RT-qPCR assay and immunofluorescence assay using an anti-ORF2 antibody, which has been reported earlier (10). The number of ORF2 positive cells was counted in 5–10 independent fields, and the percentage was calculated with reference to the number of 4',6-diamidino-2-phenylindole (DAPI) positive cells. Values are plotted as mean (+SD). g3-HEV-Luc-replicon encodes the G-Luc gene in place of the ORF2 region (63). Its activity was measured from cell culture media using the *Renilla* luciferase assay kit, following the manufacturer’s instructions (Promega, Wisconsin, USA). The G-Luc values were normalized to that of cell viability and plotted as mean (+ SD) of three independent experiments done in triplicate.

### Purification of g1-HEV clinical isolate and infection of Huh7 cells

A fecal sample was obtained from an acute HEV patient (with informed consent), admitted to the Gastroenterology Department of All India Institute of Medical Sciences, New Delhi, India, following the institutional guidelines. The fecal sample was resuspended in phosphate-buffered saline (10% [wt/vol]) and centrifuged at 100,000 g for 1 hour at 4°C. The collected supernatant was passed through a 0.2 µm filter. The viral suspension was concentrated by tangential flow filtration using a 30 kDa disposable cassette. The final concentrated virus suspension was filtered through a 0.2 µm filter in a biosafety cabinet, and aliquots were stored at −80°C. Total RNA was isolated from an aliquot of the viral suspension, and RT-qPCR was performed, along with standards prepared from known quantities of the pSK-HEV-2 plasmid. HEV genomic RNA was quantified from the standard curve, and the genome copy number of the virus was estimated using the following formula: number of copies = ng × (number/mole)/bases × (ng/g) × (g/mole of bases), as described earlier (26). A total of 4 × 10^5^ Huh7 cells were infected with 1.8 × 10^4^ genome copies of the g1-HEV clinical isolate. After a 1-hour infection, cells were washed three times in Phosphate Buffered Saline (PBS) and maintained for the indicated periods in complete medium. Viral replication was measured by RT-qPCR and immunofluorescence assay, as described above.

### Statistical analysis

Data are presented as mean + SD of three independent experiments. *P*-values were calculated by a two-tailed Student’s *t*-test (paired two samples for means). A *P*-value < 0.05 was considered statistically significant.

## Data Availability

The RNA-seq data used in this study are available in GEO database (accession no. GSE135619).

## SUPPLEMENTAL MATERIAL

Figure S1 msystems.00438-25-s0001.tif g3-HEV RNA level in Huh7 cells.

Figure S2 msystems.00438-25-s0002.tif g1-HEV RNA level in Huh7 cells.

Figure S3 msystems.00438-25-s0003.tif g1-HEV RNA level in Huh7 cells.

Figure S4 msystems.00438-25-s0004.tif g1-HEV RNA level in Huh7 cells.

Figure S5 msystems.00438-25-s0005.tif Antiviral effect of etravirine on HEV.

Table S1 msystems.00438-25-s0006.docx Modulators.

Table S2 msystems.00438-25-s0007.xlsx Network property analysis of DPN.

ASM does not own the copyrights to Supplemental Material that may be linked to, or accessed through, an article. The authors have granted ASM a non-exclusive, world-wide license to publish the Supplemental Material files. Please contact the corresponding author directly for reuse.
