# Supplementary figures and images for: Network controllability analysis reveals the antiviral potential of Etravirine against hepatitis E virus infection
Source: mSystems. 2025 Aug 15;10(9):e00438-25. doi: 10.1128/msystems.00438-25 (PMC12456023; doi:10.1128/msystems.00438-25)

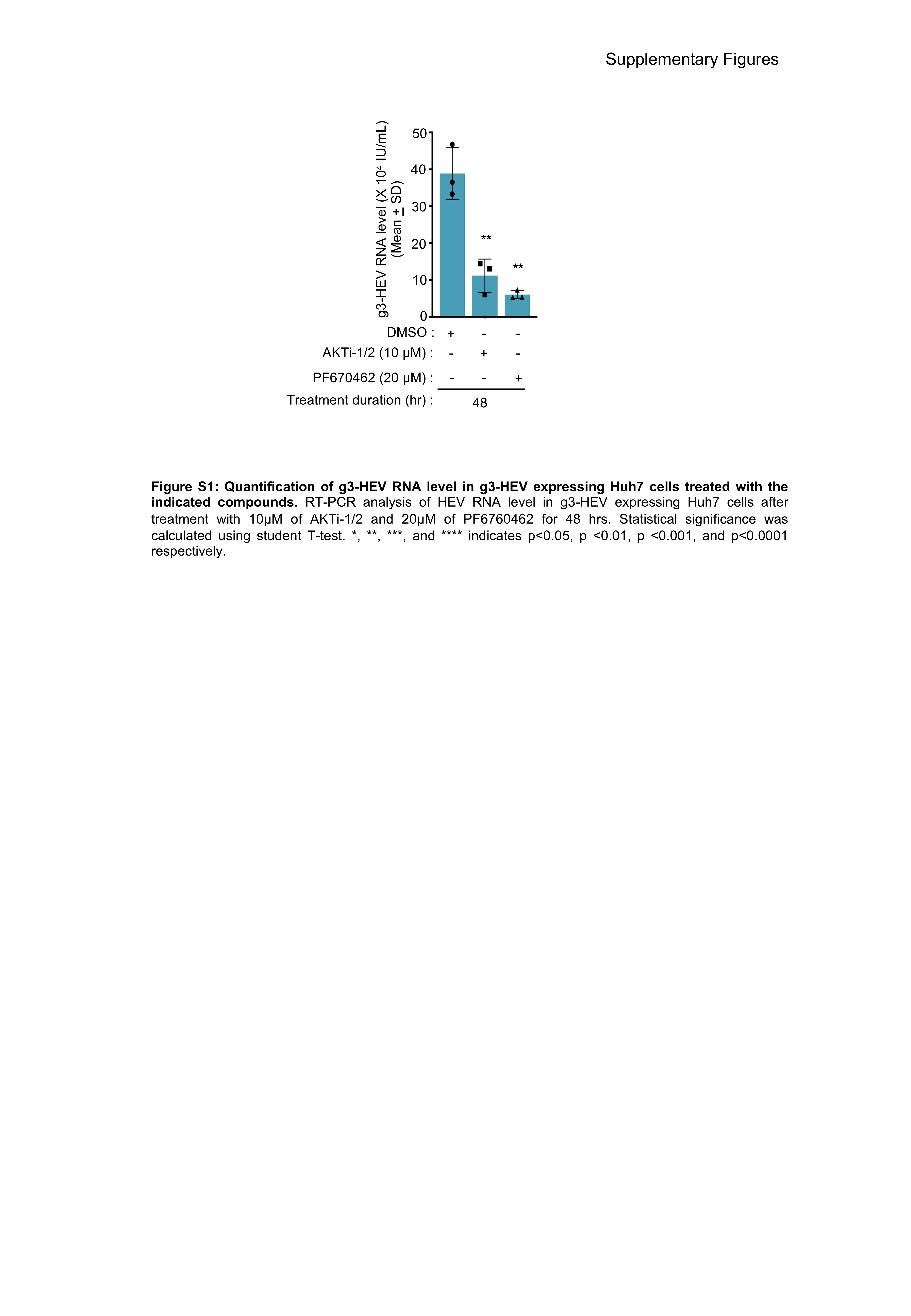

Supplement: Figure S1 — g3-HEV RNA level in Huh7 cells. [file msystems.00438-25-s0001.tif]

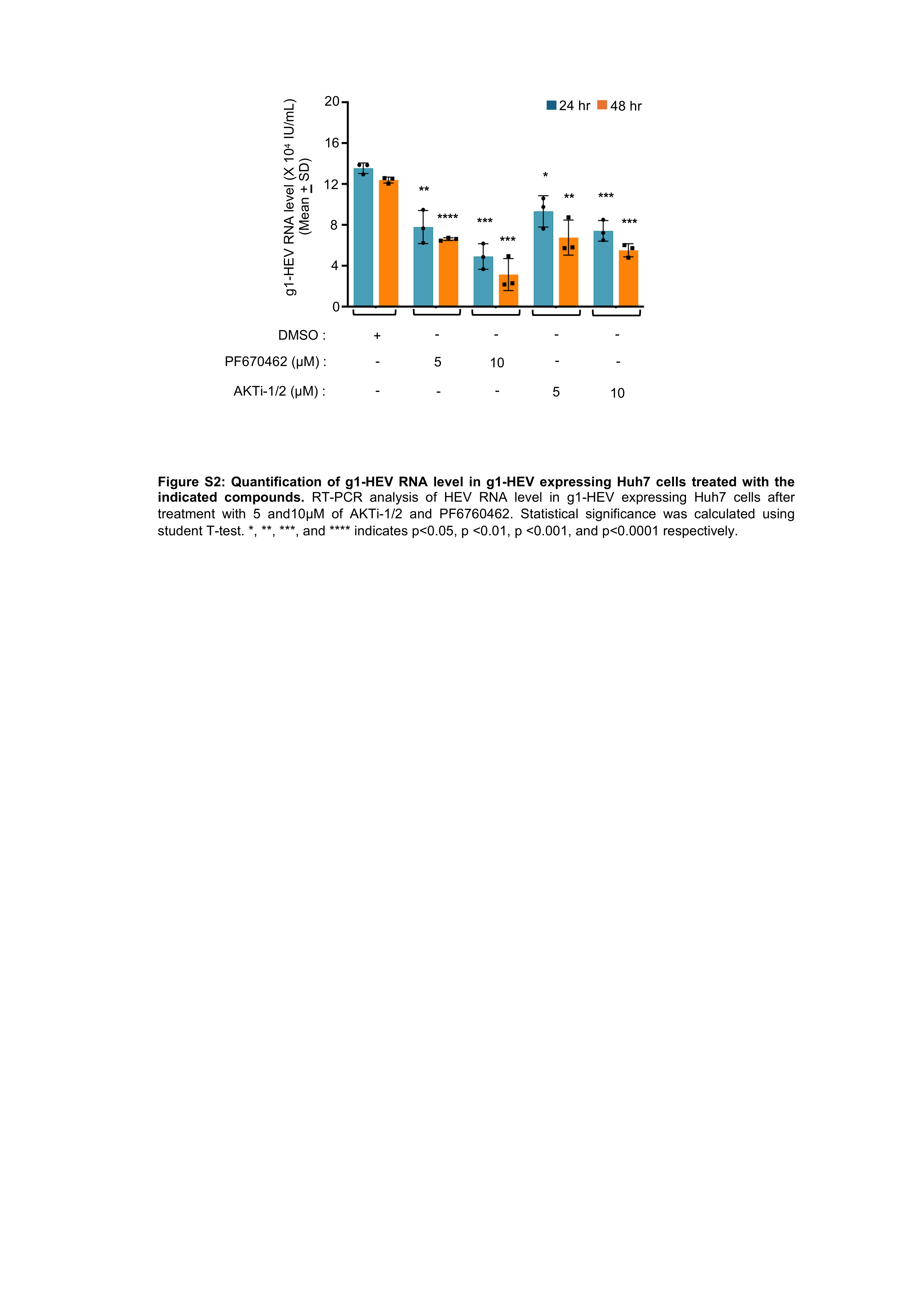

Supplement: Figure S2 — g1-HEV RNA level in Huh7 cells. [file msystems.00438-25-s0002.tif]

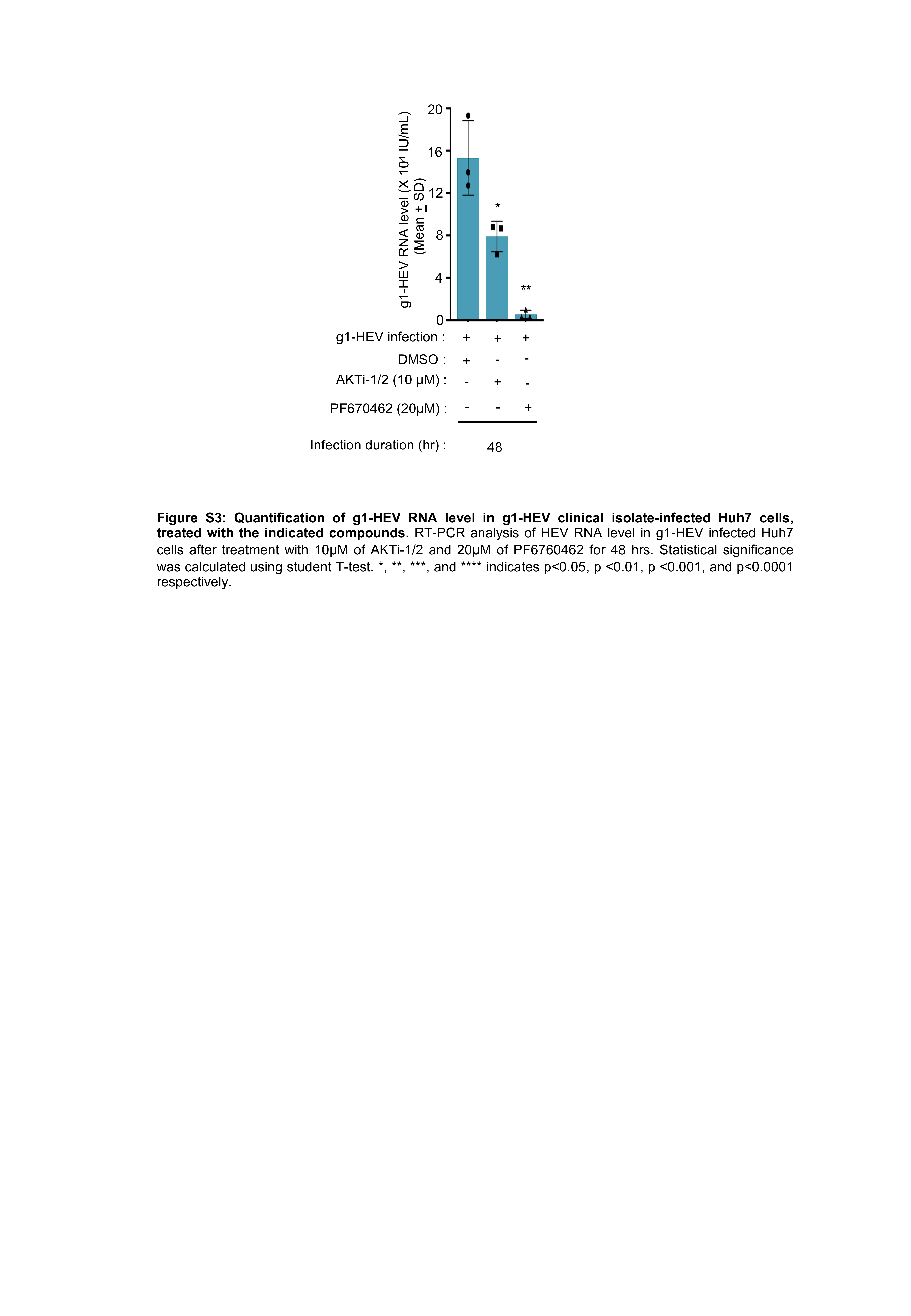

Supplement: Figure S3 — g1-HEV RNA level in Huh7 cells. [file msystems.00438-25-s0003.tif]

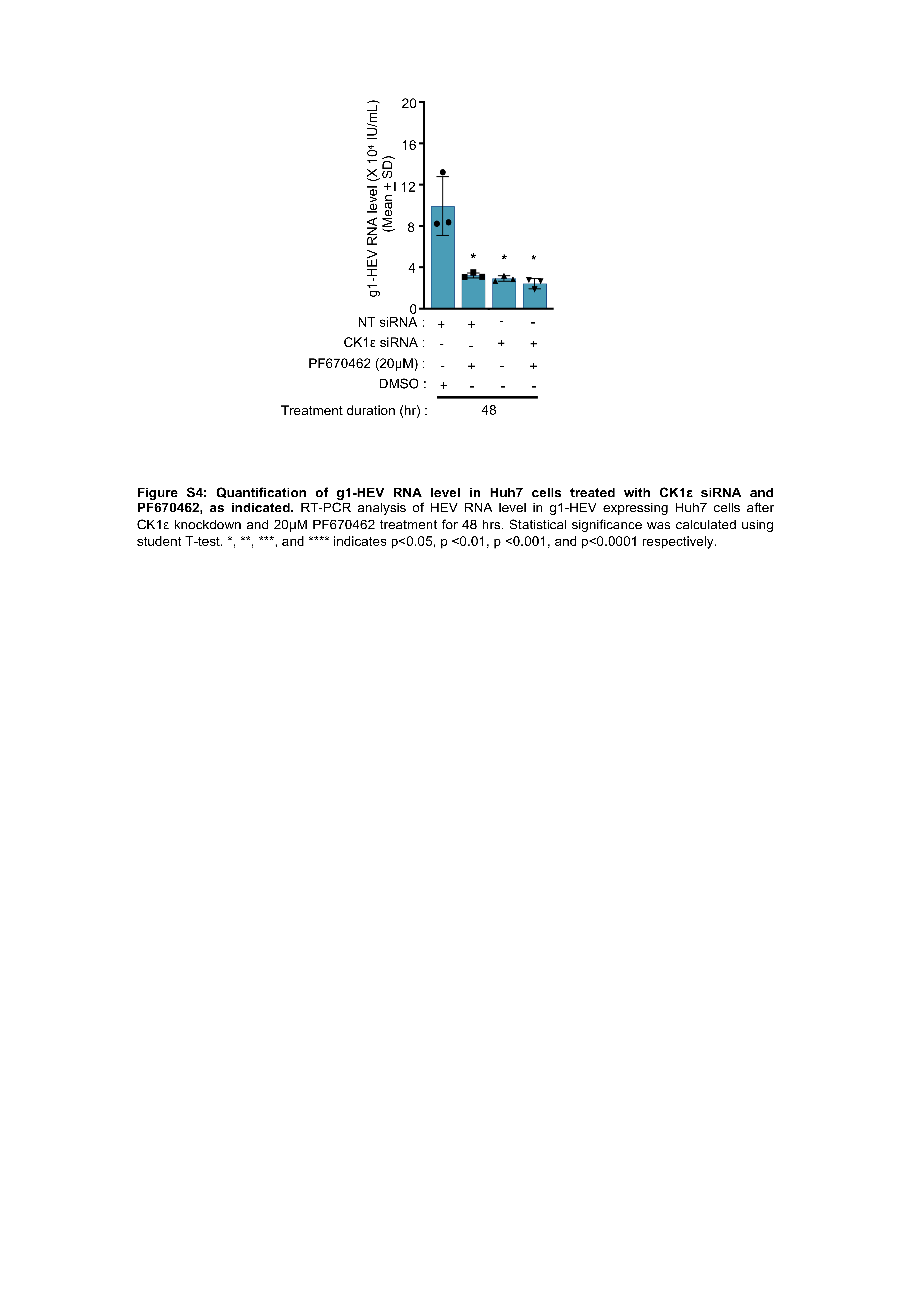

Supplement: Figure S4 — g1-HEV RNA level in Huh7 cells. [file msystems.00438-25-s0004.tif]

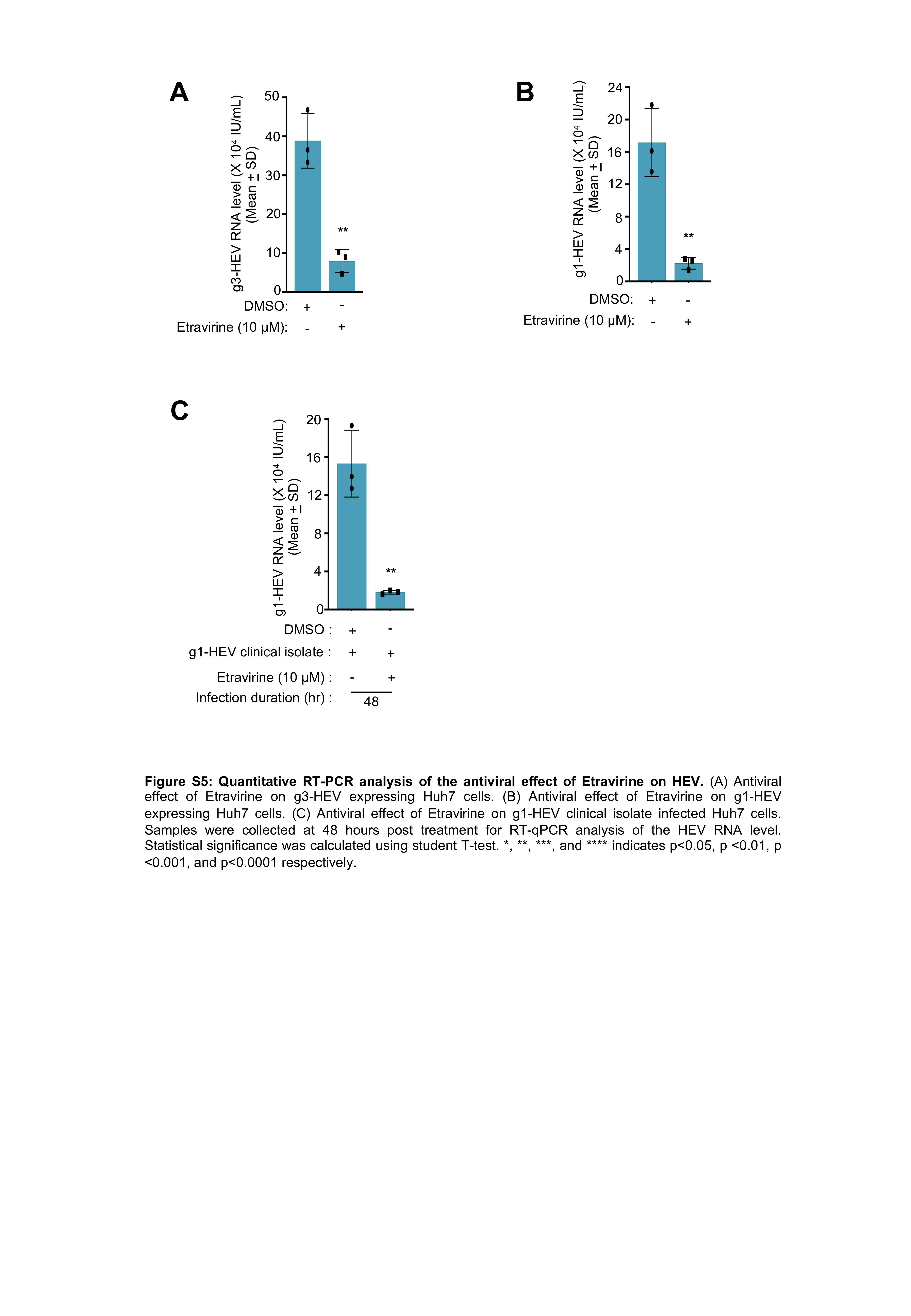

Supplement: Figure S5 — Antiviral effect of etravirine on HEV. [file msystems.00438-25-s0005.tif]
