# Supplementary material for: Network controllability analysis reveals the antiviral potential of Etravirine against hepatitis E virus infection
Source: mSystems. 2025 Aug 15;10(9):e00438-25. doi: 10.1128/msystems.00438-25 (PMC12456023; doi:10.1128/msystems.00438-25)
Supplement: Table S1 — Modulators. [file msystems.00438-25-s0006.docx]

Table S1. List of modulators.

| **Uniprot Id** | **Gene Name** |
| --- | --- |
| P30291 | WEE1 |
| P32320 | CDA |
| P26358 | DNMT1 |
| Q07869 | PPARA |
| P29274 | ADORA2A |
| P30542 | ADORA1 |
| P29275 | ADORA2B |
| P0DMS8 | ADORA3 |
| Q6P093 | AADACL2 |
| Q8TDU6 | GPBAR1 |
| Q7Z2Z2 | EFL1 |
| P21462 | FPR1 |
| P08729 | KRT7 |
| P16083 | NQO2 |
| P49674 | CSNK1E |
| P48729 | CSNK1A1 |
| P48730 | CSNK1D |
| P78368 | CSNK1G2 |
| P49354 | FNTA |
| P49356 | FNTB |
| P50052 | AGTR2 |
| P08069 | IGF1R |
| P31749 | AKT1 |
| P05771 | PRKCB |
| P06748 | NPM1 |
| P28335 | HTR2C |
| P28222 | HTR1B |
| P28566 | HTR1E |
| P30939 | HTR1F |
| P28223 | HTR2A |
| P41595 | HTR2B |
| P34969 | HTR7 |
| P08908 | HTR1A |
| P28221 | HTR1D |
| P47898 | HTR5A |
| P50406 | HTR6 |
